# Molecular Profile of Important Genes for Radiogenomics in the Amazon Indigenous Population

**DOI:** 10.3390/jpm14050484

**Published:** 2024-04-30

**Authors:** Milena Cardoso de Lima, Cinthia Costa de Castro, Kaio Evandro Cardoso Aguiar, Natasha Monte, Giovanna Gilioli da Costa Nunes, Ana Caroline Alves da Costa, Juliana Carla Gomes Rodrigues, João Farias Guerreiro, Ândrea Ribeiro-dos-Santos, Paulo Pimentel de Assumpção, Rommel Mario Rodríguez Burbano, Marianne Rodrigues Fernandes, Sidney Emanuel Batista dos Santos, Ney Pereira Carneiro dos Santos

**Affiliations:** 1 Oncology Research Center, Federal University of Pará, Belém 66073-005, PA, Brazil; lcmilena98@gmail.com (M.C.d.L.); cinthiaconstadecastro@gmail.com (C.C.d.C.); kaio.evandro@hotmail.com (K.E.C.A.); ntshmonte@gmail.com (N.M.); giliolicnunes@gmail.com (G.G.d.C.N.); carolinecostaodonto19@gmail.com (A.C.A.d.C.); julianacgrodrigues@gmail.com (J.C.G.R.); joao.guerreiro53@gmail.com (J.F.G.); assumpcaopp@gmail.com (P.P.d.A.); rommelburbano@gmail.com (R.M.R.B.); fernandesmr@yahoo.com.br (M.R.F.);; 2 Laboratory of Human and Medical Genetics, Federal University of Pará, Belém 66075-110, PA, Brazil; akelyufpa@gmail.com

**Keywords:** radiogenomics, radiotherapy, genetic variants, exome, indigenous population, oncology

## Abstract

Radiotherapy is focused on the tumor but also reaches healthy tissues, causing toxicities that are possibly related to genomic factors. In this context, radiogenomics can help reduce the toxicity, increase the effectiveness of radiotherapy, and personalize treatment. It is important to consider the genomic profiles of populations not yet studied in radiogenomics, such as the indigenous Amazonian population. Thus, our objective was to analyze important genes for radiogenomics, such as *ATM*, *TGFB1*, *RAD51*, *AREG*, *XRCC4*, CDK1, *MEG3*, *PRKCE*, *TANC1*, and *KDR*, in indigenous people and draw a radiogenomic profile of this population. The NextSeq 500^®^ platform was used for sequencing reactions; for differences in the allelic frequency between populations, Fisher’s Exact Test was used. We identified 39 variants, 2 of which were high impact: 1 in *KDR* (rs41452948) and another in *XRCC4* (rs1805377). We found four modifying variants not yet described in the literature in *PRKCE*. We did not find any variants in *TANC1*—an important gene for personalized medicine in radiotherapy—that were associated with toxicities in previous cohorts, configuring a protective factor for indigenous people. We identified four SNVs (rs664143, rs1801516, rs1870377, rs1800470) that were associated with toxicity in previous studies. Knowing the radiogenomic profile of indigenous people can help personalize their radiotherapy.

## 1. Introduction

Radiotherapy (RT) is one of the most important treatments for cancer. It is used in more than half of cancer patients and can have a curative or palliative goal by reducing the rate of local recurrence and improving overall survival. In RT, the tumor tissue is irradiated with ionizing radiation, which can penetrate the adjacent healthy tissue and cause toxic effects there, as the radiation damages the DNA [1,2]. In this sense, the toxic effects, adverse effects caused by the intensity of the dose, lead to losses both for the individual and due to the poor quality of life. Particular attention must be paid to childhood cancers, as these can be long-lasting, with correspondingly high impacts of radiotherapy and on the healthcare system due to the increase in public expenditure [1,3].

Radiotoxicity (RTX) may vary depending on the tissue irradiated [1]. Mucositis, xerostomia, subcutaneous fibrosis, and dysphagia [4] occur with squamous cell carcinoma; esophagitis may occur with radiation to the lung [5]. RT to the chest can cause pain, fibrosis, upper limb edema, pericarditis, and ischemic and valvular heart disease [6]. RT in the prostate can lead to dermatitis, urinary incontinence, and bladder and erectile dysfunction [7].

Radiosensitivity is a tendency to develop adverse effects in tissues under the effects of cell death due to radiation; this generates resistance to treatment and is influenced by biological or genetic factors, for example, tumor heterogeneity [8,9]. It is understood that radiosensitivity driven by genetic variation is one of the fundamental factors for radiotoxicity [10]. Given this, radiogenomics searches for biomarkers of the response to radiotherapy have been used to personalize treatments among individuals undergoing radiotherapy according to their radiogenomic profile [3,10].

RT dosing follows the guidelines of the Radiation Therapy Oncology Group, which has established the same standards for the same tumor sites. However, due to individual differences, some doses may be excessive or insufficient [11]. This scenario may be even more complicated when considering the profile of genetically diverse populations such as indigenous populations, due to their process of geographic isolation and to genetic and evolutionary factors such as genetic drift, founder effects, natural selection, and inbreeding [12].

Therefore, it is pertinent to analyze important genes related to radiogenomics in the Amazonian indigenous population, to obtain information regarding the genomic profile of this population regarding the response to radiotherapy and, in the future, to obtain pertinent results through the control of mutations. Thus, the objective of the study was to analyze the exome of the *ATM*, *TGFB1*, *RAD51*, *AREG*, *XRCC4*, CDK1, *MEG3*, *PRKCE*, *TANC1*, and *KDR* genes in the indigenous population and draw a genomic profile of this population, which has been poorly studied with regard to radiogenomics.

We emphasize that the Brazilian population is one of the most heterogeneous in the world, marked by indigenous, African, and European ancestry; the indigenous population contributes significantly to the formation of Latin American groups, with a share of about 30, especially in the northern region of Brazil [13,14]. In addition, studies conducted with indigenous peoples from other countries have shown high rates of cancer deaths [15]. On the other hand, there have been few genetic studies aiming to understand oncologic mechanisms and therapies that have dealt with this group. We can also point out that there have been no studies evaluating the impact of variants important for radiogenomics on the indigenous population and the population mixed with it.

## 2. Materials and Methods

### 2.1. Population Analysis for the Study

The study participants and their ethnic leaders signed the Free and Informed Consent Form. This study was approved by the National Ethics and Research Commission (CONEP) and the Research Ethics Committee of the Center for Tropical Medicine of the Federal University of Pará (UFPA) under CAAE 20654313.6.0000.5172. The population is made up of 64 indigenous people from the Amazon region of Brazil, representing 12 ethnicities: Asurini do Xingu, Arara, Araweté, Asurini do To-cantins, Awagujá, Kayapó/Xikrin, Zo’é, Wajãpi, Karipuna, Phurere, Munduruku, and Ju-runa. The population was healthy and did not undergo radiotherapy. The genetic ancestry of the population group was obtained through a panel of 61 ancestry informative markers (AIMs), which were used to identify individual ancestry and a mixture of three continents (European, African, and Amerindian). The results obtained were associated with other world populations, according to information available in version 3 of the 1000 Genomes database (available online at http://www.1000genomes.org, accessed on 25 October 2023) and the Exome Aggregation Consortium (ExAC). These populations are composed of 661 Africans (AFR), 346 Americans (AMR), 504 East Asians (EAS), 503 Europeans (EUR), and 489 South Asians (SAS).

### 2.2. DNA Extraction and Exome Analysis

The method used for DNA extraction was phenol–chloroform extraction [16]. Verification of the integrity of the sample was carried out using a NanoDrop 8000 spectrophotometer, and tracing was performed using the Nextera Rapid Capture Exome (Illumina^®^, San Diego, CA, USA) and SureSelect Human All Exon V6 kits (Agilent, Santa Clara, CA, USA). The NextSeq 500^®^ platform (Illumina^®^, San Diego, CA, USA) was used for sequencing reactions using the NextSeq 500 High-output v2 300 cycle kit (Illumina^®^, San Diego, CA, USA).

### 2.3. Selection of Genes and Variants

The first criterion for variant consideration was 10 minimum coverage values, and then the impact of each variant was considered according to the SNPeff v 4.3 classification (https://pcingola.github.io/SnpEff/; accessed on 20 February 2024). In addition, we considered only variants with a significant allelic frequency in at least three continental populations. As a result of the exome analysis, 143 variants were found. After selection based on the above criteria, 39 variants remained to be followed up in this study.

Ten genes were selected for this study based on their association with radiotherapy efficacy and/or toxicity; some have been included in previous studies and are indexed in databases such as PubMed. The genes are described in Table 1.

### 2.4. Bioinformatics Analysis

FASTQ was used to analyze the quality of the reads (FastQCv.0.11—https://www.bioinformatics.babraham.ac.uk/projects/fastqc/; accessed on 20 February 2024), and filters were applied to the samples in order to disregard low-quality reads (fastx_tools v.0.13—http://hannonlab.cshl.edu/fastx_toolset/; accessed on 20 February 2024). The reference genome (GRCH38) was used to map and align the samples, using BWA v.0.7 (http://bio-bwa.sourceforge.net/; accessed on 20 February 2024). The file was indexed and classified according to the alignment generated by the reference genome (SAMtools v.1.2—http://sourceforge.net/projects/samtools/; accessed on 20 February 2024). From there, the alignment was processed to remove PCR duplication (Picard Tools v.1.129—http://broadinstitute.github.io/picard/; accessed on 20 February 2024) and to perform structuring quality readjustment and local realignment (GATK v. 3.2—https://www.broadinstitute.org/gatk/; accessed on 20 February 2024). Then, the results were processed to establish the reference genome variants (GATK v.3.2). ViVa1 (Viewer of Variants) software (v1)—developed by the bioinformatics team at the Federal University of Rio Grande do Norte (UFRN)—was used to analyze the variant annotations. The databases employed for variant annotations were SnpEff v.4.3., Ensembl Variant Effect Predictor (Ensembl version 99), and ClinVar (v.2018-10). For in silico predictions of pathogenicity, SIFT (v.6.2.1), PolyPhen-2 (v.2.2), LRT (November 2009), Mutation Evaluator (v.3.0), and Mutation Tester (v. 2.0) were used. FATHMM (v.2.3), PROVEAN (v.1.1.3), MetaSVM (v1.0), M-CAP (v1.4), and FATHMM-MKL (http://fathmm.biocompute.org.uk/about.html; accessed on 20 February 2024) were also used. More information about bioinformatics analysis is given in the works of Rodrigues et al. [37] and Ribeiro-dos-Santos et al. [38].

### 2.5. Statistical Analysis

Fisher’s Exact Test was used to determine statistically significant differences (*p* value ≤ 0.05) between the frequencies of the variants in the world populations (AFR, EUR, AMR, EAS, and SAS). The allele frequencies of the variants found in the study population were determined by allele enumeration. Comparisons between the allele frequencies of the variants found in the indigenous population and in the five world populations were performed using Fisher’s Exact Test. To avoid discrepancies due to different population sizes, we leveled all variant frequencies with the size of the indigenous population. The frequencies of the five populations were taken from Phase 3 of the 1000 Genomes Project (http://www.1000genomes.org; accessed on 20 February 2024) and the Exome Aggregation Consortium (ExAC). These allele frequencies were also used to calculate the dissimilarity matrix and to generate the multidimensional scaling (MDS). The analyses were performed with RStudio v. 3.5.1.

## 3. Results

**Figure 1:**
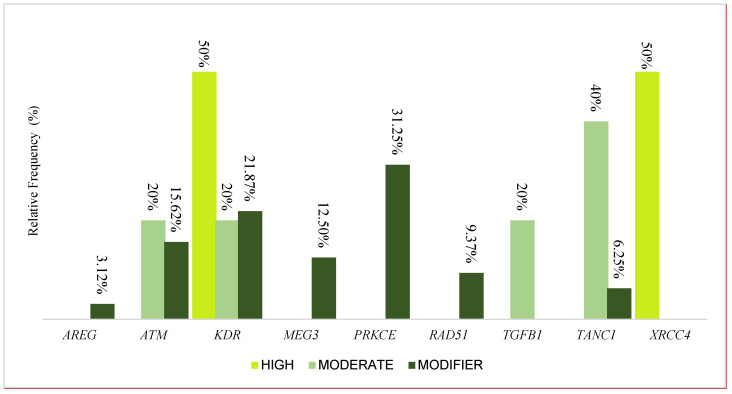
The relative contributions of variants discriminated according to high, modifier, or moderate impact in the *AREG*, *ATM*, *KDR*, *MEG3*, *PRKCE*, *RAD51*, *TGFB1*, *TANC1*, and *XRCC4* genes.

From the analyses, we identified 39 variants distributed across the 9 genes: 1 belonging to the *AREG* gene, 6 to the *ATM* gene, 9 to the *KDR* gene, 4 to the *MEG3* gene, 10 to the *PRKCE* gene, 3 to the *RAD51* gene, 1 to the *TGFB1* gene, 4 to the *TANC1* gene, and 1 to the *XRCC4* gene. Figure 1 presents the distribution of variants according to their high, modifier, or moderate impact; thus, 2 were classified as high-impact, 28 as modifiers, and 5 as moderate.

Two high-impact variants were identified, distributed in the *KDR* and *XRCC4* genes; for the modifying variants, 3.12% (1) were in the *AREG* gene, 15.62% (5) in *ATM*, 21.87% (7) in *KDR*, 12.50% (4) in *MEG3*, 31.25% (10) in *PRKCE*, 9.37% (3) in *RAD51*, and 6.25% (2) in *TANC1*. Regarding the moderate variants, *ATM*, *KDR*, and *TGFB1* each had one moderate variant, while two were in the *TANC1* gene. The variants are described in Table 2 with information about the ID, the impact predicted by SNPeff software v 4.3, the region, the type, the nucleotide change, and the frequency presented in world populations. The low-impact variants are presented in Supplementary Table S1.

As for the variants not yet described in the literature, four were found, all in the *PRKCE* gene on chromosome 2; these are listed in Table 3 and are characterized by a single nucleotide change and an insertion/deletion. The variants are sequentially located at 45652078, 45652096, 45652087, and 45652092. Of these variants, three had a frequency of 0.0833 in the indigenous population and one had a frequency of 0.01677. We intend to conduct further studies to further explore the effects of these variants on protein activity to obtain additional information on radiogenomics in a poorly studied population.

Of the variants described in Table 2, rs41452948 and rs1805377 are of high impact, the first occurring in the *KDR* gene and the second in the *XRCC4* gene, the first on chromosome 4 and the second on chromosome 5. Both are represented by a change in nucleotide from G to A. These variants showed a significant allelic frequency in the indigenous population compared to the AFR, AMR, EUR, and SAS populations and a high, modifying, and moderate impact, as shown in Table 3 Comparisons with low-impact variants can be found in Supplementary Table S2.

In addition, 43 variants showed a nonzero allelic frequency in at least three global populations. However, the differences in frequency in the five continental populations compared to the indigenous population were significant for only 18 of these alleles, as described in Table 4.

**Figure 2:**
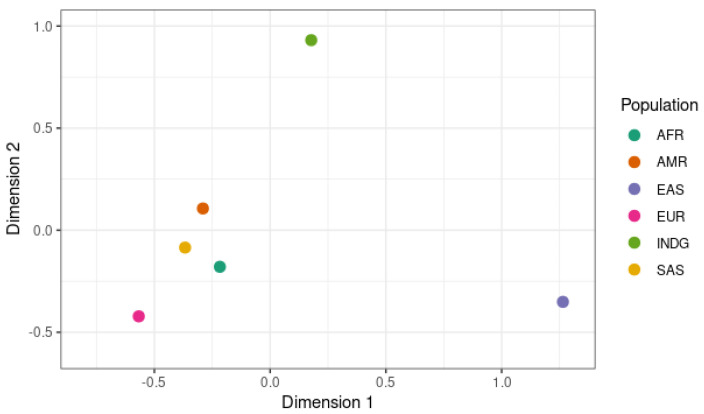
Differences in allele frequencies of the variants studied in the continental populations and indigenous population, plotted on an MDS.

Figure 2 shows the different genotypes in the global population and the indigenous population on a multidimensional scale (MDS) determined using Fisher’s Exact Test. Looking at the genes selected for this exome, our results show that the indigenous population (INDG) has a genetic profile that differs significantly from those of the five world populations, especially those of East Asians (EAS) and Africans (AFR).

## 4. Discussion

The curative potential of radiotherapy is limited by the intrinsic radioresistance of tumor cells, which is related to the heterogeneity of the tumor and the surrounding microenvironment, as well as to various genetic alterations [39]. In addition to radioresistance, patients may experience clinically significant side effects, namely, acute reactions such as erythema, follicular reaction, pruritus, moist or dry desquamation, ulceration, and necrosis, or delayed reactions such as tissue fibrosis, atrophy, firm subcutaneous tissue, and subcutaneous swelling [40,41].

GWA studies are often used to identify genetic variants associated with complex and multifactorial diseases [42,43]. GWA studies linking radiogenomics to radiotherapy toxicity show that genetic variants, treatment variables, and other clinical factors are independent predictors of radiotoxicity. This contributes to the suggestion that common variants may improve traditional models of the likelihood of complications in normal tissues [44,45]. When DNA is exposed to radiation, DNA double-strand breaks occur, which favors a response to this damage, such as cellular radioresistance [46,47]. This was the first exome study to examine genes associated with the response to radiotherapy in indigenous populations, a group that is usually underrepresented in genetic studies, especially in precision medicine [48].

In our results, we found 39 variants with nonzero allele frequencies in the study population, 4 of which have never been found in other genetic databases and none of which are in the CDK1 gene. Therefore, of the 35 variants present in all five populations, we highlight two high-impact variants, rs41452948 in the *KDR* gene and rs1805377 in the *XRCC4* gene, as presenting statically significant differences when comparing the indigenous population with African, American, European, and South Asian populations, as shown in Table 4.

We emphasize that many variants were identified in intronic regions, where they were believed not to encompass gene functions. However, intronic regions play an important role in genome regulation. For example, they influence the maturation of mRNA and transcriptional regulation through alternative splicing, which can directly influence a gene’s response to radiotoxicity [49]. The rs1805377 in the splice site acceptor + intron could have consequences such as disruption of the normal splicing process, leading to important changes in parts of the final mRNA messenger gene, which, in turn, could cause genetic disorders or disease, depending on the gene affected and the specific type of variant [50].

The variants found only in the indigenous population are all located in the *PRKCE* gene, which is described in the literature as a gene associated with the response to radiation [27]. Members of the PKC family phosphorylate a variety of protein targets and are involved in cell signaling pathways [26]. These variants have a modifying clinical effect; three of the variants had an allelic frequency of 0.0833 in indigenous people and one had a frequency of 0.1667. These mutations with a modifying effect could be potential markers for indigenous populations in the Amazon region. However, we emphasize the intention to conduct further studies to consolidate the knowledge of these potential new variants.

Polymorphisms in the *RAD51* gene have been associated in previous studies with symptoms of heart failure and the appearance of a new primary tumor in patients with breast cancer treated with trastuzumab and RT [6], suggesting an important gene for RTX; however, such polymorphisms were not detected in the population studied. Other studies have also investigated variations in DNA repair genes—such as *ATM*, *RAD51*, and *XRCC4*—that cause differences in the response to radiation between individuals. These differences are characterized by increased toxicity, which normally emanates from irradiated tissue [51], but these variants were also not detected in the Indigenous population, suggesting protection from these toxicities.

We discovered four variants in the *TANC1* gene, two with a moderate impact (rs34588551, rs4664277) and two with a modifying impact in the intronic region (rs34344829, rs146371641). In our analyses, we found a statistically significant difference regarding rs146371641 in all five world populations compared to the indigenous group (Table 4), which is related to the fact that *TANC1* is an important gene for radiogenomics; this could show that the indigenous population has a different profile in the use of radiotherapy.

*TANC1* plays a crucial role in the recruitment of fusion-capable myoblasts during myotube formation. In addition, expression of this gene has been found in various tissues such as adipose tissue and the adrenal cortex [3,31]. *TANC1* was associated with late radiotherapy toxicity in a large genomic study cohort with a European population. The results suggested that this gene is involved in muscle damage processes induced by radiation. However, in our study, the variants presented in the European study were not found, which may be explained by the genomic difference between the population originating from Brazil and the European population [37]. The influencing variant rs664143, located in the *ATM* intron, showed significant differences in the five world populations. This variant showed a worse prognosis in patients with squamous cell carcinoma of the esophagus and non-small cell lung cancer after radiotherapy. In addition, the A allele was associated with shorter survival, while the G allele indicated an increased risk of disease progression [52].

The rs1801516 variant, which has a moderate impact on *ATM*, showed significant differences in the indigenous population compared to the American, European, and South Asian populations (see Table 4). This variant has been studied with regard to the effects after radiotherapy, with results suggesting that individuals with the minor allele have an increased risk of developing late fibrosis after radiotherapy [53]. In studies by McDuff et al. [54], Andreassen et al. [55], and Kerns et al. [45], the variant was associated with the risk of general toxicity, acute toxicity, late toxicity, acute skin toxicity, acute rectal toxicity, telangiectasia, and fibrosis.

The variant with a moderate impact on the *KDR* gene, rs1870377, showed a significant difference, especially when comparing the indigenous and European populations (Table 4). A study by Tinhofer et al. [56] showed that despite the poorer prognosis of patients with squamous cell carcinoma of the head and neck, the A > T allele of germline variant rs1870377 was associated with longer survival in risk groups of patients with stage IV squamous cell carcinoma of the oropharynx and hypopharynx who underwent chemotherapy. On the other hand, Butkiewicz et al. [57] analyzed 422 patients with squamous cell carcinoma of the head and neck undergoing radiotherapy and concluded that VEGFR2 rs1870377 TT is a significant borderline risk factor for lower local recurrence and an independent predictor of poor prognosis.

When analyzing the toxicity of the rs1870377 variant in patients undergoing 5-fluorouracil-based chemotherapy for locally advanced rectal cancer, the VEGFR2 H472Q Q/Q genotype was associated with a higher risk of grade 3 mucositis of the proximal upper gastrointestinal tract in arm 2 of the study (induction and concomitant prolonged intravenous infusion of 5-FU with radiotherapy). However, in arm 1 (bolus of 5-FU followed by prolonged intravenous infusion of 5-FU with radiotherapy), this genotype was associated with a lower risk of mucositis [22].

The variant with a moderate influence on the *TGFB1* gene, rs1800470, showed statistically significant differences between the five global populations and the indigenous population (Table 4). According to Xiao Y et al. [58], the T allele of variant rs1800470 (CT/TT) is associated with the risk of developing radiation-induced pneumonia in patients with esophageal squamous cell carcinoma. In patients with oropharyngeal squamous cell carcinoma undergoing radiotherapy, the presence of the rs1800470 variant of TGFβ1 may reduce and modify the risk of death and recurrence [59].

Previous research also found mitigating differences between the genetic profile of indigenous peoples and those of the other five world populations examined here [60,61,62]. However, the physical and geographic isolation of these peoples contributes to a gap in epidemiologic and genetic information. Therefore, knowledge of their molecular profile may be of importance to indigenous populations, as well as those intermixed with them [63].

In the European world, the applications of radiogenomics concepts are more advanced, considering studies such as those by O’Sullivan, NJ; Kelly [64], which used models to predict metastases after neoadjuvant radiotherapy and showed how these concepts can be used to identify low-risk patients in order not to subject them to interventions such as tumor dissection. In addition, radiogenomics has also been used to predict genetic mutations in colorectal cancer to optimize the outcome of radiotherapy and enable targeted therapy [65].

## 5. Conclusions

This study investigated the presence of genetic variants involved in the process of radiotherapy toxicity in an indigenous Amazonian population. No variants already associated with late RTX were found. We found two high-impact variants and four variants that have not yet been described in the other five world populations examined and may be exclusive to this population. Future research on therapeutic approaches targeting indigenous and admixed populations is critical.

Personalized medicine research has gained much attention for its role in targeting therapies and making predictions. This can help doctors plan more appropriate treatment regimens, reduce radiation damage in high-risk patients, and optimize outcomes in low-risk patients.

## Figures and Tables

**Table 1:** The functions of the *AREG*, *ATM*, *KDR*, *MEG3*, *PRKCE*, *RAD51*, *TGFB1*, *TANC1*, and *XRCC4* genes.

Gene	Description
*AREG*	Related to epidermal growth factor (EGF) and transforming growth factor alpha (TGF-alpha). The protein interacts with the EGF/TGF-alpha receptor, promotes the growth of normal epithelial cells, and inhibits the growth of carcinoma cell lines. Studies indicate that this gene is involved in tumor escape and radiation resistance and is significantly overexpressed after radiotherapy [17].
*ATM*	Favors the interruption of the cell cycle, senescence, and apoptosis and acts on the cellular response to DNA double-strand breaks, occasionally caused by radiotherapy. Therefore, inhibiting ATM seems to increase the effectiveness of radiotherapy [18]. SNPs (rs620815 and rs11212570) in the gene are associated with an increased risk of gastrointestinal toxicity and odynophagia in individuals treated with radiotherapy [19].
*CDK1*	Cyclin-dependent kinase (CDK1), a cell cycle regulator, is intimately involved in several cellular events vital for cell survival, such as the regulation of gene expression. CDK1 is responsible for regulating the transition between the G2 phase and mitosis [20]. CDK1 is inactivated when DNA damage occurs due to ionizing irradiation to arrest the cell cycle at the G2 checkpoint and facilitate the repair of double-strand breaks, which may be beneficial in radiotherapy treatment [21].
*KDR*	The kinase insert domain receptor, also known as VEGFR (vascular endothelial growth factor), acts to promote angiogenesis in normal and pathological conditions, such as cancer [22]. An SNP (rs1870377) in the gene was associated with mucositis in patients treated with chemoradiotherapy [23].
*MEG3*	A tumor suppressor that negatively regulates proteins such as p53 and STAT3, consequently affecting cell proliferation and metastasis and promoting apoptosis [24,25]. The rs1032552 variant of *MEG3* has been associated with toxicities such as dermatitis and anemia in individuals treated with chemoradiotherapy [25].
*PRKCE*	Members of the PKC family phosphorylate a wide variety of protein targets and are involved in cellular signaling pathways such as neuron channel activation and apoptosis [26]. Furthermore, the literature indicates that the *PRKCE* gene is also associated with radiation toxicity in lung cancer [27].
*RAD51*	Acts on DNA repair and, when there is interference in the binding to ATPase and single-stranded DNA, generates genomic instability [4,28]. Gene expression is more frequent in certain tumor tissues and implies a lower survival rate, tumor progression, immunosuppression, radioresistance, and worse prognosis [29,30]. It is associated with radiotoxicity in HER2-positive breast cancer [6].
*TANC1*	Tetratricopeptide repeat, ankyrin repeat, and coiled-coil containing 1 is a protein-coding gene, considered a scaffold component related to the regulation post-synapse [31]. It is involved in the repair of damaged muscle cells, suggesting that its biological mechanism is associated with the greater development of radiation toxicity; this is due to its potential role in the regeneration of radio-induced damage in muscle tissue [32].
*TGFB1*	Growth factor β 1 acts to control growth, proliferation, differentiation, and apoptosis. It appears to be a marker of radiosensitivity, where its reduction during radiotherapy treatment is associated with a positive response [33]. It plays an important role in inflammation and cell proliferation, which may be related to radiation-induced fibrosis [34].
*XRCC4*	Acts in the repair of DNA double-strand breaks [35]. Studies have indicated that reducing *XRCC4* expression delays the repair of DNA damage. Furthermore, the protein is a radiosensitivity marker and a promising study object for radiogenomics [36].

**Table 2:** Descriptions of the variants in the *AREG*, *ATM*, *KDR*, *MEG3*, *PRKCE*, *RAD51*, *TGFB1*, *TANC1*, and *XRCC4* genes according to their impact, region, variant type, and change in nucleotide.

Impact	Gene	dbSNP	Region	Var Type	Change in Nucleotide	Frequencies					
						INDG	AFR	AMR	EAS	EUR	SAS
High	*KDR*	rs41452948	Protein_Structural_Interaction_ Locus	SNV	G > A	0.0517	0	0	0.004	0	0
High	*XRCC4*	rs1805377	Splice_Site_Acceptor+Intron	SNV	G > A	0.6667	0.476	0.333	0.704	0.140	0.172
Modifier	*AREG*	rs368667736	Intron	SNV	C > T	0.1000	0.226	0.242	0.522	0.145	0.242
Modifier	*ATM*	rs672655	Intron	SNV	A > G	0.1250	0.216	0.640	0.420	0.617	0.626
Modifier	*ATM*	rs58978479	Intron	INDEL	AT > A	0.2459	0.005	0.025	0.030	-	0.030
Modifier	*ATM*	rs2066734	Intron	INDEL	TAA > T	0.6230	0.123	0.532	0.384	0.451	0.549
Modifier	*ATM*	rs664143	Intron	SNV	A > G	0.2105	0.700	0.683	0.447	0.627	0.679
Modifier	*ATM*	rs3218681	Intron	INDEL	AA > AAA	0.0000	0.430	0.643	0.408	0.625	0.667
Modifier	*MEG3*	rs142677044	Intragenic	SNV	G > C	0.1897	0	0.045	0.014	0	0
Modifier	*MEG3*	rs139003317	Intron	INDEL	CCCT > C	0.0541	0.606	0.173	0.001	0.210	0.182
Modifier	*MEG3*	rs56363527	Intron	SNV	C > T	0.0571	0.061	0.321	0.237	0.335	0.276
Modifier	*MEG3*	rs7160821	Intragenic	SNV	G > A	0.1905	0.216	0.461	0.325	0.344	0.333
Modifier	*KDR*	rs2305946	Intron	SNV	C > T	0.0135	0.104	0.140	0.454	0.238	0.152
Modifier	*KDR*	rs2219471	Intron	SNV	T > C	0.0469	0.135	0.147	0.459	0.240	0.121
Modifier	*KDR*	rs3816584	Intron	SNV	A > G	0.0135	0.103	0.140	0.454	0.238	0.152
Modifier	*KDR*	rs7655964	Intron	SNV	A > C	0.6406	0.108	0.496	0.313	0.363	0.306
Modifier	*KDR*	rs17085310	Intron	SNV	G > A	0.2344	0.001	0.112	0.074	0.005	0.055
Modifier	*KDR*	rs3214870	Intron	INDEL	GG > GGG	0.0635	0.197	0.146	0.453	0.239	0.152
Modifier	*KDR*	rs7692791	Intron	SNV	C > T	0.0857	0.390	0.464	0.630	0.545	0.721
Modifier	*RAD51*	rs45455000	Intron	SNV	T > G	0.0000	0.116	0.081	0.133	0.075	0.126
Modifier	*PRKCE*	rs60465117	Intron	SNV	A > C	0.0000	0.066	0.017	0.085	0.005	0.084
Modifier	*PRKCE*	rs4953294	Intron	SNV	G > A	0.0270	0.067	0.183	0.087	0.267	0.337
Modifier	*PRKCE*	rs1987070	Intron	SNV	C > A	0.0139	0.152	0.141	0.219	0.273	0.197
Modifier	*PRKCE*	rs201731045	Intron	SNV	T > C	0.0806	0.003	0.001	0	0	0
Modifier	*PRKCE*	rs2249505	Intron	SNV	C > T	0.6471	0.014	0.398	0.584	0.216	0.195
Modifier	*PRKCE*	rs10495929	Intron	SNV	G > A	0.0147	0.186	0.133	0.059	0.149	0.215
Modifier	*TANC1*	rs34344829	Intron	SNV	A > G	0.0270	0.023	0.287	0.048	0.513	0.186
Modifier	*TANC1*	rs146371641	Intron	INDEL	CCC > CCCC	0.0405	0.185	0.295	0.048	0.514	0.186
Modifier	*RAD51*	rs45457497	Intron	SNV	T > G	0.5000	0.275	0.393	0.644	0.153	0.342
Modifier	*RAD51*	rs200723181	Intron	INDEL	T > TCT	0.0000	0.301	0.341	0.569	0.150	0.268
Moderate	*ATM*	rs1801516	Non_Synonymous_Coding	SNV	G > A	0.0000	0.008	0.097	0.016	0.162	0.080
Moderate	*KDR*	rs1870377	Non_Synonymous_Coding	SNV	T > A	0.0469	0.090	0.131	0.465	0.235	0.149
Moderate	*TANC1*	rs34588551	Non_Synonymous_Coding	SNV	C > T	0.0323	0.030	0.249	0.003	0.347	0.122
Moderate	*TANC1*	rs4664277	Non_Synonymous_Coding	SNV	A > G	0.9453	0.461	0.610	0.618	0.373	0.535
Moderate	*TGFB1*	rs1800470	Non_Synonymous_Coding	SNV	G > A	0.2143	0.586	0.494	0.445	0.618	0.554

(-) No annotation. INDG: Indigenous Amazonian population, AFR: African population, AMR: American population, EAS: East Asian population, EUR: European population, SAS: South Asian population.

**Table 3:** Description of new variants found in the indigenous population from the Brazilian Amazon in genes relevant to radiotherapy.

Gene	Chromosome	Position	Var Type	Region Detailed	Reference	Variant	Impact	Protein Change	Variant Allele Frequency
*PRKCE*	chr2	45652078	SNV	5UTR	G	C	MODIFIER	c.-23G > C	0.0833
*PRKCE*	chr2	45652096	INDEL	5UTR	C	CCCCCCAGGGT	MODIFIER	c.-5_-4insCCCCCAGGGT	0.0833
*PRKCE*	chr2	45652087	SNV	5UTR	T	C	MODIFIER	c.-14T > C	0.0833
*PRKCE*	chr2	45652092	SNV	5UTR	G	C	MODIFIER	c.-9G > C	0.1667

**Table 4:** Comparison between the allelic frequencies in Indigenous Amazonian (INDG) and continental populations (AFR, AMR, EAS, EUR, and SAS) described in the 1000 Genomes and the Exome Aggregation Consortium (ExAC).

GENE	dbSPN	INDG × AFR *	INDG × AMR *	INDG × EAS *	INDG × EUR *	INDG × SAS *
*PRKCE*	rs2249505	2.17 × 10^−29^	2.40 × 10^−4^	-	2.58 × 10^−11^	1.18 × 10^−12^
*KDR*	rs2305946	1.06 × 10^−2^	5.43 × 10^−4^	1.23 × 10-^17^	1.56 × 10^−7^	2.88 × 10^−4^
*TANC1*	rs34344829	6.22 × 10^−9^	6.81 × 10^−20^	-	-	5.17 × 10^−5^
*KDR*	rs3816584	1.06 × 10^−2^	5.43 × 10^−4^	1.23 × 10^−17^	1.56 × 10^−7^	2.88 × 10^−4^
*TANC1*	rs34588551		1.07 × 10^−6^	-	1.83 × 10^−10^	1.50 × 10^−2^
*KDR*	rs2219471	4.08 × 10^−2^	1.72 × 10^−2^	2.51 × 10^−14^	2.82 × 10^−5^	-
*PRKCE*	rs60465117	1.21 × 10^−2^	-	1.62 × 10^−3^	-	1.62 × 100^−3^
*PRKCE*	rs4953294	-	1.00 × 10^−4^	-	5.65 × 10^−8^	6.14 × 10^−11^
*TANC1*	rs146371641	5.63 × 10^−4^	7.86 × 10^−8^	-	6.18 × 10^−18^	5.63 × 10^−4^
*MEG3*	rs142677044	1.31 × 10^−7^	1.50 × 10^−3^	1.08 × 10^−5^	1.31 × 10^−7^	1.31 × 10^−7^
*MEG3*	rs139003317	1.90 × 10^−21^	9.17 × 10^−3^	2.28 × 10^−2^	7.49 × 10^−4^	5.70 × 10^−3^
*KDR*	rs7655964	3.81 × 10^−18^	3.55 × 10_-_^2^	7.25 × 10^−7^	2.92 × 10^−5^	3.75 × 10^−7^
*TANC1*	rs4664277	1.51 × 10^−17^	2.25 × 10^−10^	4.70 × 10^−10^	2.36 × 10^−22^	6.04 × 10^−14^
*KDR*	rs17085310	1.31 × 10^−9^	2.04 × 10^−2^	8.51 × 10^−4^	2.07 × 10^−8^	1.49 × 10^−4^
*PRKCE*	rs1987070	2.88 × 10^−4^	5.43 × 10^−4^	6.57 × 10^−7^	4.13 × 10^−9^	5.43 × 10^−6^
*PRKCE*	rs201731045	3.12 × 10^−3^	3.12 × 10^−3^	3.12 × 10^−3^	3.12 × 10^−3^	3.12 × 10^−3^
*XRCC4*	rs1805377	6.73 × 10^−3^	7.42 × 10^−7^	-	4.44 × 10^−17^	6.88 × 10^−15^
*KDR*	rs3214870	4.60 × 10^−3^	-	1.63 × 10^−12^	2.22 × 10^−4^	-
*MEG3*	rs56363527	-	1.44 × 10^−7^	1.49 × 10^−4^	3.89 × 10^−8^	7.28 × 10^−6^
*KDR*	rs7692791	3.91 × 10^−8^	5.38 × 10^−11^	9.11 × 10^−20^	4.71 × 10^−15^	3.10 × 10^−25^
*MEG3*	rs7160821	-	1.25 × 10^−5^	2.34 × 10^−2^	1.21 × 10^−2^	1.72 × 10^−2^
*PRKCE*	rs10495929	1.08 × 10^−5^	1.00 × 10^−3^	-	2.88 × 10^−4^	6.57 × 10^−7^
*AREG*	rs368667736	1.79 × 10^−2^	8.23 × 10^−3^	1.05 × 10^−12^	-	8.23 × 10^−3^
*KDR*	rs1870377	-	4.08 × 10^−2^	1.05 × 10^−14^	5.17 × 10^−5^	1.72 × 10^−2^
*KDR*	rs41452948	2.28 × 10^−2^	2.28 × 10^−2^	-	2.28 × 10^−2^	2.28 × 10^−2^
*ATM*	rs672655	-	6.04 × 10^−17^	4.11 × 10^−7^	1.17 × 10^−15^	4.38 × 10^−16^
*ATM*	rs1801516	-	8.12 × 10^−4^	-	1.21 × 10^−6^	3.12 × 10^−3^
*ATM*	rs58978479	9.43 × 10^−9^	4.53 × 10^−7^	2.11 × 10^−6^	-	2.11 × 10^−6^
*TGFB1*	rs1800470	5.18 × 10^−9^	1.07 × 10^−5^	2.45 × 10^−4^	2.48 × 10^−10^	8.16 × 10^−8^
*RAD51*	rs45455000	1.02 × 10^−4^	3.12 × 10^−3^	2.39 × 10^−5^	3.12 × 10^−3^	4.99 × 10^−5^
*ATM*	rs2066734	4.38 × 10^−16^	-	3.84 × 10^−4^	1.43 × 10^−2^	-
*ATM*	rs664143	1.45 × 10^−14^	2.64 × 10^−13^	2.45 × 10^−4^	1.13 × 10^−10^	2.64 × 10^−13^
*RAD51*	rs45457497	6.74 × 10^−4^	-	4.70 × 10^−2^	2.40 × 10^−8^	2.52 × 10^−2^
*ATM*	rs3218681	4.41 × 10^−19^	5.56 × 10^−32^	7.45 × 10^−18^	6.51 × 10^−0^	1.17 × 10^−33^
*RAD51*	rs200723181	7.85 × 10^−13^	1.05 × 10^−14^	3.03 × 10^−27^	5.43 × 10^−6^	5.36 × 10^−11^

(-) No annotation. INDG: Indigenous Amazonian population, AFR: African population, AMR: American population, EAS: East Asian population, EUR: European population, SAS: South Asian population. * *p*-value ≤ 0.05.

## Data Availability

Data are contained within the article and Supplementary Materials.

## Supplementary Materials

The following support information can be downloaded from: https://www.mdpi.com/article/10.3390/jpm14050484/s1. Table S1: Descriptions of low-impact *AREG*, *ATM*, *KDR*, *MEG3*, *PRKCE*, *RAD51*, *TGFB1*, *TANC1*, and *XRCC4* gene variants, as well as allele frequencies in continental populations (African (AFR), American (AMR), East Asian (EAS), European (EUR), and South Asian (SAS)) described in the 1000 genome database. Table S2: Paired comparisons (*p*-values) with significant results of allele frequencies in the Amazonian indigenous population (INDG) and in the continental populations (African (AFR), American (AMR), East Asian (EAS), European (EUR), and South Asian (SAS)) described in the 1000 genome database.
