# Supplementary material for: Molecular Profile of Important Genes for Radiogenomics in the Amazon Indigenous Population
Source: J Pers Med. 2024 Apr 30;14(5):484. doi: 10.3390/jpm14050484 (PMC11122349; doi:10.3390/jpm14050484)
Supplement: Supplementary file 1 [file jpm-14-00484-s001.zip › jpm-2950154-supplementary.pdf]

SUPPLEMENTARY TABLE 1

Gene	dbSPN	Impact	Region			INDG
TANC1	rs2288110	LOW	SYNONYMOUS_CODING	SNV	A>C	0.9211
TANC1	rs3821296	LOW	SYNONYMOUS_CODING	SNV	A>G	0.9375
TANC1	rs4664276	LOW	SYNONYMOUS_CODING	SNV	C>T	0.9375
XRCC4	rs1056503	LOW	SYNONYMOUS_CODING	SNV	T>G	0.6905
MEG3	rs151191249	LOW	SPLICE_SITE_REGION+INTRON	SNV	G>C	0.2266
PRKCE	rs1143692	LOW	SYNONYMOUS_CODING	SNV	C>T	0.0000
TANC1	rs2288105	LOW	SPLICE_SITE_REGION+INTRON	SNV	C>T	0.0800
PRKCE	rs1143691	LOW	SYNONYMOUS_CODING	SNV	C>T	0.2581

AFR	AMR	EAS	EUR	SAS
0.351	0.601	0.615	0.372	0.535
0.349	0.602	0.616	0.372	0.535
0.395	0.604	0.616	0.372	0.535
0.476	0.333	0.706	0.140	0.172
0.004	0.052	0.017	0.015	0.004
0.004	0.052	0.007	0.069	0.077
0.182	0.261	0.455	0.084	0.171
0.006	0.121	0.396	0.095	0.238

SUPPLEMENTARY TABLE 2						
Gene	dbSPN	Frequencies				
		INDG x AFR	INDG x AMR	INDG x EAS	INDG x EUR	INDG x SAS
TANC1	rs2288110	2.08E-21	9.95E-04	2.47E-08	5.41E-20	6.12E-12
TANC1	rs3821296	5.98E-23	4.36E-10	1.97E-09	1.60E-21	3.04E-13
TANC1	rs4664276	3.68E-20	4.36E-10		1.60E-21	3.04E-13
XRCC4	rs1056503	1.93E-03	1.03E-07		2.11E-18	3.52E-16
MEG3	rs151191249	4.24E-08	2.58E-04	3.27E-07	3.27E-07	4.24E-08
PRKCE	rs1143692		2.28E-02		6.36E-03	3.12E-03
TANC1	rs2288105	3.68E-02	4.12E-04	3.16E-11		
PRKCE	rs1143691	2.00E-09	1.09E-02	3.59E-02	1.81E-03	
